# Triple Vascular Compression in an Adolescent: A Report of a Rare Case

**DOI:** 10.7759/cureus.93630

**Published:** 2025-10-01

**Authors:** Mário Andrade, Cristiana Azevedo, Marta Pinto Dias, Ana Sofia V Gomes

**Affiliations:** 1 Family Medicine, Unidade Local de Saúde de Santo António, Porto, PRT

**Keywords:** may-thurner syndrome, nutcracker syndrome (ncs), primary care, superior mesenteric artery syndrome, surgery

## Abstract

Superior mesenteric artery syndrome (SMAS), or Wilkie’s syndrome, is a rare cause of intestinal obstruction due to duodenal compression between the aorta and superior mesenteric artery (SMA). It is often linked to weight loss and anatomical variations that reduce the mesenteric fat pad, worsening the compression. Delayed diagnosis can lead to severe complications, including malnutrition. Similarly, nutcracker syndrome (NCS) and May-Thurner syndrome (MTS) involve vascular compressions that may result in serious complications if not promptly managed.

A 14-year-old previously healthy adolescent developed progressive postprandial abdominal pain and vomiting for six months, leading to severe weight loss. This led to multiple emergency visits for dehydration, requiring IV fluids and hospitalization. Symptoms worsened after a growth spurt, causing malnutrition. A CT scan confirmed SMAS, alongside NCS and MTS. Due to renal impairment, he required prolonged hospitalization with parenteral nutrition and psychological support. Symptoms recurred at 16 and 18 years old, leading to further hospitalizations. Given his poor quality of life and failed conservative treatment, a gastrojejunostomy was performed at 18 years old, resulting in a favorable outcome.

This case highlights the diagnostic challenges of SMAS and its frequent association with vascular compression syndromes. Early recognition of persistent vomiting, weight loss, and dehydration is crucial. A multidisciplinary approach, including nutritional, surgical, and psychological support, optimizes outcomes. Primary care physicians play a key role in early diagnosis, referral, and long-term management to prevent complications and improve quality of life.

## Introduction

Superior mesenteric artery syndrome (SMAS), also known as Wilkie’s syndrome, is a rare cause of proximal intestinal obstruction. It is characterized by the compression of the third portion of the duodenum between the abdominal aorta and the superior mesenteric artery (SMA), often secondary to the reduction of the mesenteric fat pad. SMAS has an estimated incidence of 0.13% to 0.3%, with a predilection for slender young females [1]. The main clinical manifestations include epigastric pain, nausea, postprandial vomiting (initially with food content and later bilious), early satiety, postprandial discomfort, and weight loss, which may mimic anorexia. It is associated with congenital anatomical variants such as a reduced aortomesenteric angle and frequently follows significant weight loss [2]. Diagnosis requires correlating the clinical presentation with radiological findings such as duodenal dilation and a narrowed aortomesenteric angle [3], typically via computed tomography (CT), which remains the gold standard imaging modality. The disease course is often insidious, with progressive worsening of symptomatology. Clinical improvement is common with prone or left lateral decubitus and with forward trunk flexion [4]. Initial management is usually conservative, focusing on nutritional support and weight restoration [5]. Surgical intervention, such as Strong’s procedure, gastrojejunostomy, or duodenojejunostomy, is indicated in refractory cases.

Nutcracker syndrome (NCS) refers to compression of the left renal vein (LRV), most commonly between the SMA and the aorta (anterior NCS), or less frequently between the aorta and the vertebral column (posterior NCS). It is also a rare vascular anomaly, typically presenting with flank pain, with or without hematuria, proteinuria, or renal impairment. The aetiology is usually anatomical [6]. Diagnosis is based on a combination of clinical symptoms and radiological findings, although venous pressure gradients may not always correlate with disease severity [7]. Management ranges from conservative monitoring to surgical intervention in severe cases [8].

May-Thurner syndrome (MTS) is caused by compression of the left common iliac vein by the overlying right common iliac artery and the lumbar vertebral body. While often asymptomatic, symptomatic cases carry significant morbidity, particularly due to deep vein thrombosis and post-thrombotic syndrome. It primarily affects females aged between 30 and 60 years, particularly those using combined oral contraceptives. Symptomatic patients may present with unilateral left lower limb swelling, venous claudication, or venous hypertension, often relieved by limb elevation [9].

Although these syndromes are individually rare, the co-occurrence is exceedingly uncommon. This report describes a unique case of an adolescent diagnosed with all three entities.

## Case presentation

We report the case of a 14-year-old boy with no relevant medical history, from a single-parent household with a stable family dynamic. He developed recurrent non-radiating abdominal pain and persistent vomiting, initially with food content and later with bilious characteristics. These symptoms progressively worsened, resulting in significant weight loss and multiple episodes of dehydration, leading to several emergency department (ED) visits. On one occasion, hospitalization was required due to acute pre-renal kidney injury.

**Figure 1:**
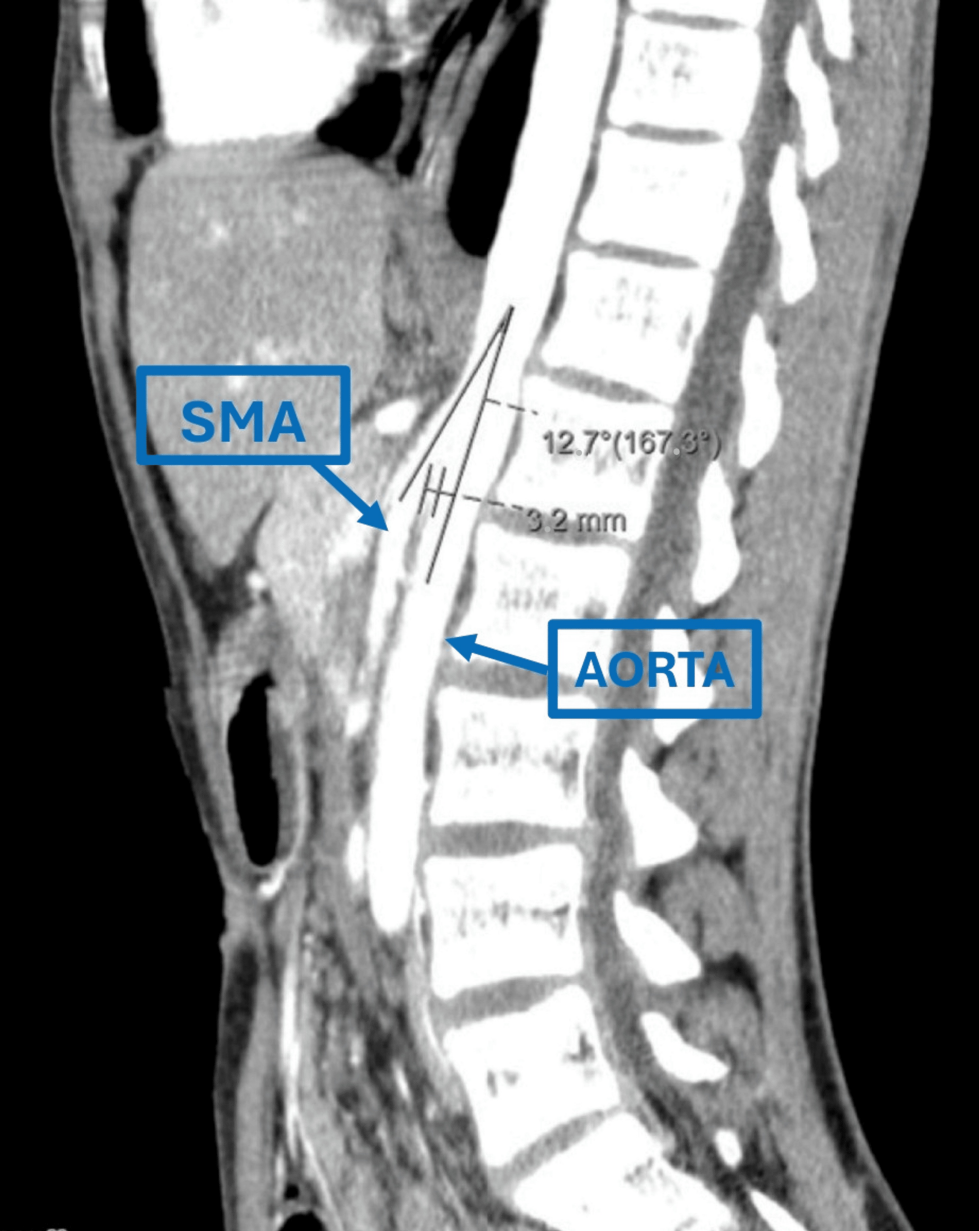
Sagittal Plane Computed Tomography (CT) Scan Sagittal plane abdominal CT scan showing superior mesenteric artery (SMA) compression of the duodenum. The aortomesenteric angle is reduced to 12.7° and the aortomesenteric distance narrowed to 3.2 mm, findings consistent with superior mesenteric artery syndrome (SMAS).

His clinical condition deteriorated over six months, coinciding with a marked growth spurt of approximately 10-12 cm within a year. He developed anorexia, asthenia, constipation, and persistent epigastric pain. On examination, he was cachectic (38.7 kg), with signs of moderate dehydration and abdominal tenderness. Laboratory investigations revealed deteriorating renal function, with a blood urea nitrogen (BUN) of 136 mg/dL (18-45 mg/dL) and a serum creatinine of 1.16 mg/dL (0.35-0.86 mg/dL), as well as proteinuria at 30 mg/dL (0-14 mg/dL). An abdominal CT confirmed SMAS, characterized by duodenal compression between the aorta and SMA (Figure 1).

**Figure 2:**
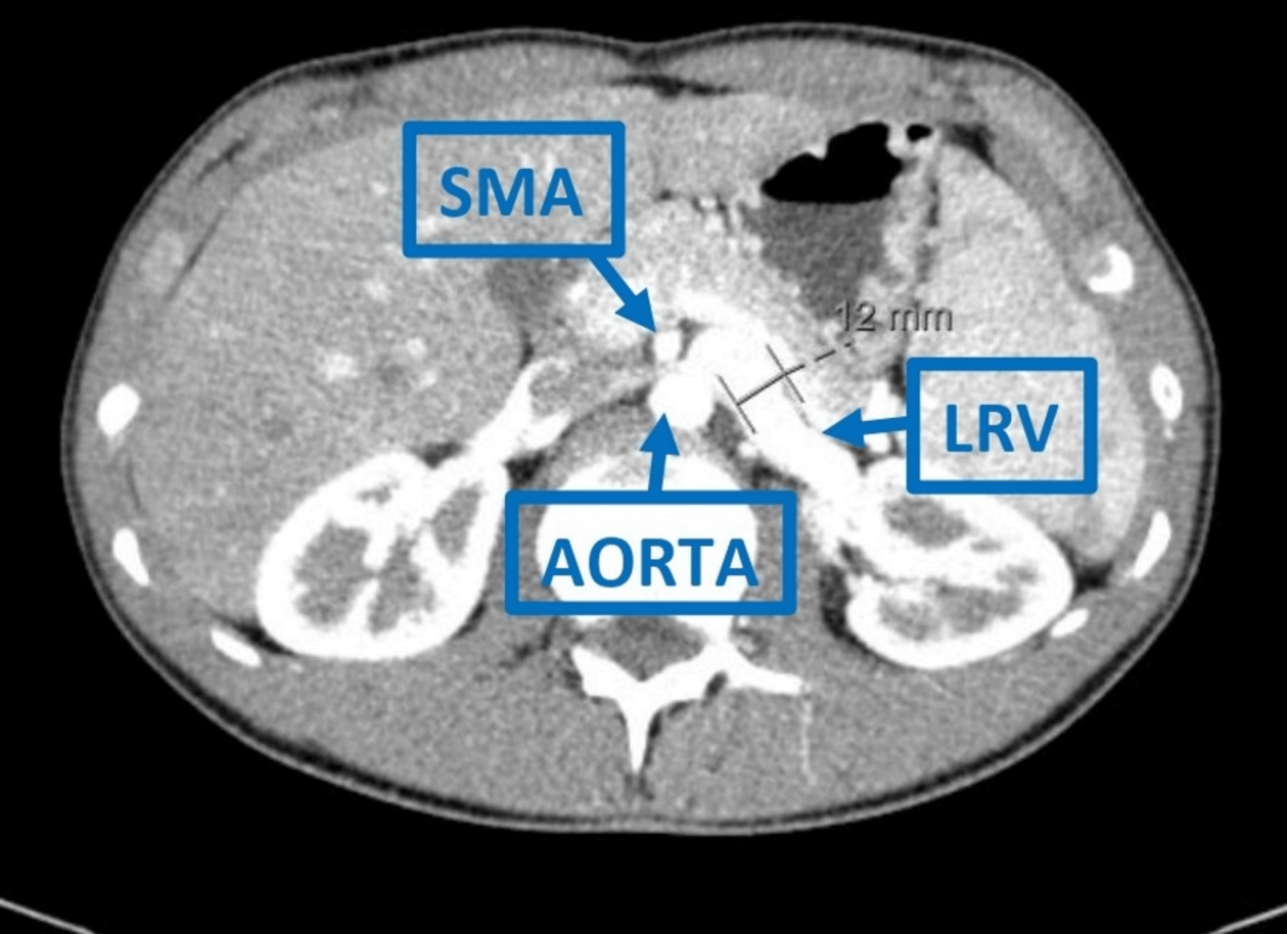
Axial Plane Computed Tomography (CT) Scan Axial plane abdominal CT scan demonstrating compression of the left renal vein (LRV) between the superior mesenteric artery (SMA) and the aorta, resulting in proximal LRV dilation. Findings are consistent with nutcracker syndrome.

**Figure 3:**
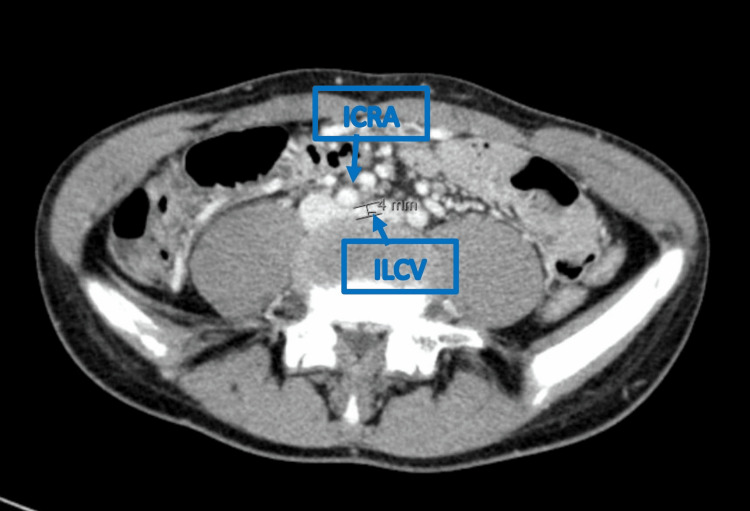
Axial Computed Tomography (CT) scan Axial abdominal CT scan demonstrating compression of the left common iliac vein (ILCV) by the overlying right common iliac artery (ICRA) against the vertebral body, resulting in venous narrowing. Findings are consistent with May-Thurner syndrome.

Concomitant diagnoses of NCS (Figure 2) and MTS (Figure 3) were also established by CT scan, further compromising his clinical status.

Due to the severity of his presentation, he required prolonged hospitalization for 67 days, needing parenteral nutrition. Oral intake was reestablished on day 21. Psychological support was integral, as he exhibited signs of depressive mood and was referred to Child and Adolescent Psychiatry. Following weight recovery to 47.6 kg and clinical stabilization, he was discharged from the hospital.

Over the subsequent two years, he remained under follow-up in Pediatric Gastroenterology and Nephrology with partial symptom resolution. At age 16, he experienced a recurrence with epigastric pain, nausea, vomiting, and weight loss of 8 kg over 45 days, requiring re-admission for nutritional support. Although symptoms initially improved with conservative measures, at age 18, he experienced recurrent episodes of severe abdominal pain, vomiting, and anorexia, leading to multiple ED admissions.

Given the recurrence of symptoms and considerable impact on quality of life, surgical management was indicated. A gastrojejunostomy was performed without complications. Four months postoperatively, he remains asymptomatic, with weight gain and no dietary restrictions.

## Discussion

This case describes a previously healthy adolescent who presented at age 14 with chronic abdominal pain, persistent vomiting, and significant weight loss. The protracted nature of his illness ultimately led to the rare diagnosis of three distinct but related vascular compression syndromes: SMAS, NCS, and MTS.

The timing of symptom onset coinciding with a growth spurt likely contributed to the depletion of retroperitoneal fat and subsequent duodenal compression, which is a common cause of the syndrome, leading to the compression that causes further weight loss and thus creates a vicious cycle [10].

The co-diagnosis of NCS introduced additional complexity, particularly regarding proteinuria and impaired renal function, requiring close nephrological follow-up. MTS may have further compromised venous return and exacerbated systemic manifestations. Those syndromes do not always have symptoms. In this case, we had renal symptoms related to NCS but no symptoms related to MTS. We always need at least a CT scan to have a diagnosis of those syndromes and relate them to clinical findings [11].

Management required prolonged inpatient care, with initiation of parenteral nutrition due to oral intolerance. It is the first-line treatment, focusing on weight regain while watching for proper caloric needs, with care for refeeding syndrome, and can cure most cases, particularly in the short term [12]. The psychological burden of chronic illness warranted specialized mental health support. Due to the failure of conservative treatment and persistent symptomatology, surgical intervention via gastrojejunostomy was undertaken, leading to a favorable outcome, even with the limited evidence on surgical interventions, due to the small number of reported cases in the literature and the absence of large clinical trials. Duodenojejunostomy is the first line of treatment [13-15]. In this case, a gastrojejunostomy was performed, which is considered a second-line option due to greater nutritional loss.

At four months postoperatively, the patient remains asymptomatic with full nutritional recovery and unrestricted oral intake. This favorable trajectory underscores the importance of timely diagnosis and the consideration of surgical management in refractory cases.

## Conclusions

SMAS presents with initially nonspecific gastrointestinal symptoms such as abdominal pain, nausea, vomiting, postprandial distress, and weight loss. Its insidious course complicates timely diagnosis. Abdominal pain with associated vomiting is a common presentation in primary care, and awareness of warning signs, including persistent or severe symptoms and weight loss, is crucial for prompt referral and diagnostic confirmation.

This case underscores the importance of considering SMAS in the differential diagnosis of chronic abdominal pain and highlights the role of early recognition, multidisciplinary management, and vigilant primary care follow-up in improving long-term outcomes. It also emphasizes the need for patient and family education to ensure adherence and prevent future recurrence.
